# Stem cell–based therapies for type 1 diabetes: Progress in differentiation, clinical translation, and immune protection

**DOI:** 10.1002/ame2.70211

**Published:** 2026-05-09

**Authors:** Zifan Li, Yu Kang, Yuyu Niu

**Affiliations:** ^1^ State Key Laboratory of Primate Biomedical Research; Institute of Primate Translational Medicine Kunming University of Science and Technology Kunming Yunnan China; ^2^ Yunnan Key Laboratory of Primate Biomedical Research Kunming Yunnan China; ^3^ Southwest United Graduate School Kunming Yunnan China

**Keywords:** immune rejection, islet organoids, stem cell therapy, type 1 diabetes, β cells

## Abstract

Transplantation of insulin‐producing cells derived from pluripotent stem cells represents a highly promising approach for the radical treatment of type 1 diabetes (T1D). Informed by a comprehensive understanding of fetal pancreatic development, directed differentiation protocol for generating pancreatic β cells from pluripotent stem cells has been established and has achieved considerable advances, enabling the production of mature, fully functional β cells that closely recapitulate the characteristics of native pancreatic β cells. Preclinical studies have shown that the transplantation of stem cell–derived islets (SC‐islets) reverses hyperglycemia in both mouse and nonhuman primate models, with a favorable safety profile. Early‐phase clinical trials have further corroborated the safety and efficacy of this approach, a subset of patients with long‐standing T1D achieved insulin independence, described as a “functional cure”, with no serious adverse events of clinical significance reported. Despite these encouraging results, substantial challenges remain. With respect to differentiation protocols, insufficient functional maturity, pronounced cellular heterogeneity, significant batch‐to‐batch variability, and the challenges of large‐scale manufacturing represent the principal unresolved limitations. Of particular concern, immune rejection remains a critical barrier even after the transplantation of autologous SC‐islets, necessitating continued reliance on immunosuppressive therapy. Cell encapsulation and gene editing strategies have emerged as potential approaches to overcome this immunological barrier. In this review, we discuss strategies for obtaining insulin‐producing cells from diverse cellular sources, summarize the latest advances in stem cell–based diabetes therapy, and propose future research directions.

## Graphical abstract

Directed differentiation of pluripotent stem cells into functional pancreatic β cells have emerged as a promising strategy for the radical treatment of type 1 diabetes, with preclinical and early clinical evidence demonstrating reversal of hyperglycemia and insulin independence. However, challenges, including incomplete functional maturity, cellular heterogeneity, and immune rejection, remain to be addressed, with cell encapsulation and gene editing representing potential solutions.

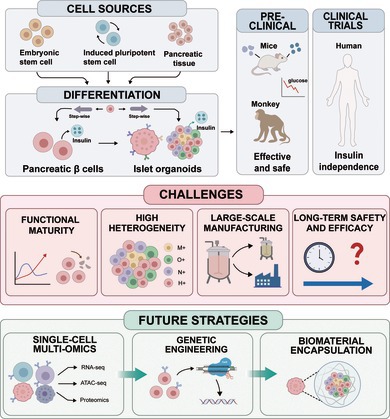

## 1 INTRODUCTION

Diabetes mellitus is a heterogenous group of metabolic diseases characterized by chronic hyperglycemia. It is divided into two major types: type 1 diabetes mellitus (T1D) is an autoimmune disease that leads to absolute insulin deficiency, and type 2 diabetes (T2D) is generally characterized by insulin resistance together with relative insulin insufficiency.^1^ The global disease burden on diabetes has consistently increased, with approximately 8.75 million individuals currently affected worldwide.^2^ Despite significant advances in continuous glucose monitors (CGM) and insulin pumps, which have greatly improved blood glucose management and quality of life in T1D patients, many patients still struggle to achieve optimal glycemic control and are prone to the risks of hyperglycemia and hypoglycemia.^3^, ^4^ Pancreas or islet transplantation is a potentially effective curative treatment of T1D, but its widespread clinical application is constrained by the critical shortage of suitable donors, and the immunosuppressive therapy necessary to keep the grafts functional is not yet overcome.^5^, ^6^

Human pluripotent stem cells (hPSCs), such as embryonic stem cells (hESCs) and induced pluripotent stem cells (hiPSCs), can proliferate self‐renew indefinitely and differentiate into any type of human cell. This renders them a viable source for the derivation of functional pancreatic β‐like cells in vitro.^7^, ^8^ Directed differentiation protocols, recapitulating the sequential signaling events of pancreatic embryogenesis, have been progressively refined, enabling the generation of stable glucose‐responsive, insulin‐secreting SC‐β cells that have since entered clinical evaluation. Nevertheless, the clinical translation and widespread application of stem cell–based therapies for diabetes are still significantly constrained by several critical challenges, the most prominent of which is immune rejection. Particularly for patients with T1D, it is necessary to target the allogeneic immune response from donor sources and avoid the recurrence of autoimmune responses against β cells.^9^ To address this challenge, cell encapsulation strategies and genetic engineering have been extensively studied. This review systematically summarizes directed differentiation protocols, animal models, clinical trials, and strategies to overcome immune rejection of hPSC‐derived β cells, presents the deficiencies and challenges of current research, and outlines future research directions, aiming to achieve safe, scalable, and immunosuppression‐free stem cell–based replacement therapy for T1D.

## 2 INSULIN‐PRODUCING CELLS FOR THE TREATMENT OF T1D

### 2.1 Functional SC‐β cells are differentiated from hPSCs

**FIGURE 1:**
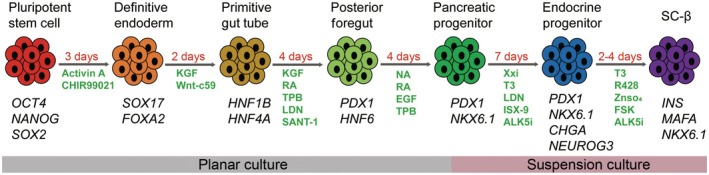
Differentiation of pancreatic β‐like cells from human pluripotent stem cells (hPSCs). This schematic illustrates the stepwise in vitro differentiation of pluripotent stem cells into pancreatic β‐like cells, recapitulating the endogenous developmental trajectory of the pancreas in vivo. Key small molecules and growth factors employed at each stage are indicated in bold green text. Italicized terms denote stage‐specific marker gene expression.

hESCs can spontaneously differentiate into insulin‐producing cells without exogenous factors.^10^, ^11^ Based on mimicking embryonic pancreatic development,^12^, ^13^ the stepwise protocol similar to that described by Lumelsky et al.^14^ has been used to induce hESCs differentiation into insulin‐producing cells.^15^ This process encompasses several key developmental stages: definitive endoderm (DE), primitive gut tube (PGT), pancreatic progenitors (PP), endocrine progenitors (EP), and hormone‐expressing endocrine cells. Furthermore, the major cytokines and critical genes in each stage are summarized in Figure 1.^16^ Through systematic screening of small molecules, the efficiency of DE differentiation has reached up to 90%,^17^, ^18^ and serum‐free differentiation systems have been successfully established.^19^ Nevertheless, these differentiation protocols predominantly yield pancreatic endoderm cells.^20^, ^21^ Although a minor proportion of insulin‐producing cells can be obtained, these cells fail to mount a dynamic response to glucose stimulation.^18^, ^22^

One of the breakthroughs comes from Pagliuca et al.^23^ in 2014; they used a combination of 11 small molecules to establish a scalable three‐dimensional (3D) suspension differentiation protocol, which can finally produce ∼40% C‐P^+^/NKX6.1^+^ glucose‐responsive SC‐β cells. Rezania et al.^24^ developed an improved seven‐step differentiation protocol, and the final product contained nearly 50% NKX6.1^+^/insulin^+^ cells. They first added vitamin C at stages 2–3 to suppress NGN3 expression and applied thyroid hormone (T3) at stage 5 to promote NKX6.1/insulin co‐expression, then identified that the AXL inhibitor R428 could induce MAFA expression. Over the ensuing decade and more, many differentiation protocols have been developed to improve the proportion of insulin‐producing cells and maturation of SC‐β cells.^25^, ^26^, ^27^, ^28^ Notably, Liu et al.^29^ established a highly efficient chemically defined protocol, which generates functional NKX6.1^+^/INS^+^ pancreatic β cells from diverse hPSC lines with a maximum efficiency of 82%. Balboa et al.^30^ developed an optimized protocol to generate functionally mature stem cell–derived islets (SC‐islets) with biphasic glucose‐stimulated insulin secretion (GSIS), organotypic cytoarchitecture, and key ion channel/exocytosis machinery analogous to primary adult islets. In addition to soluble regulators, the biophysical and biochemical properties of the extracellular matrix (ECM) have been shown to modulate stem cell differentiation,^31^, ^32^, ^33^, ^34^, ^35^ primarily by regulating cytoskeletal polymerization, recruitment of adhesion proteins, and downstream signaling cascades. Hogrebe et al.^36^ showed that modulating cell‐biomaterial interactions and actin cytoskeleton dynamics can direct pancreatic progenitor differentiation into SC‐β cells in an adhesion‐based system, producing cells with robust dynamic GSIS and rapid diabetes‐reversal capability in vivo. Although these protocols have been continuously refined, each remains associated with certain limitations. The key advantages and shortcomings of the current differentiation protocols are summarized in Table 1.

**TABLE 1:** In vitro differentiation of pluripotent stem cells into β‐cells.

Cell line	Culture	Duration	Highlights	Limitations	Year/reference
hESCs (HUES8) and iPSCs	Suspension	Five‐stage; 27–34 days	Scalable 3D differentiation of functional SC‐β cells; SC‐β cells show mature β‐cell phenotypes and GSIS function; rapid in vivo glucose‐responsive insulin secretion and ameliorates hyperglycemia in diabetic mice	Low purity of functional SC‐β cells (≈33%); transcriptome incompletely matches primary β‐cells; in vivo insulin secretion efficiency inferior to islets; lack of long‐term stability	2014/[23]
H1 and iPSCs	Planar and air–liquid interface	Seven‐stage; 27–43 days	Scalable 3D protocol for generating functional human SC‐β cells from hPSCs; achieved ~50% NKX6‐1^+^/insulin^+^ monohormonal endocrine cells; SC‐β cells recapitulate key β‐cell phenotypes (GSIS, Ca^2+^ flux, NKX6.1/PDX1 expression) and mature rapidly in vivo; reversed diabetes in ~40 days	Dynamic GSIS impaired, only 5%–10% cells show normal Ca^2+^ response; β‐cell gene expression profile incomplete; lack of long‐term stability data	2014/[24]
HUES8 cell line	Suspension	Six‐stage; >28 days	Developed serum‐free protocol via TGF‐β timing control; achieved first/s‐phase GSIS in SC‐β cells; generated high‐purity endocrine cells (≈73% C‐peptide^+^); rapidly improved glucose tolerance in diabetic mice within 10 days and function sustained up to 6 months	Insulin secretion lower than human islets; MAFA/UCN3 expression remains insufficient; performance varies across hPSC lines	2019/[27]
INS^GFP/W^ hESCs	Suspension	Seven‐stage; 26–28 days	Reaggregation generates islet‐like eBCs; robust dynamic GSIS and Ca^2+^ response; mitochondrial metabolic maturation; functions in vivo within 3 days, stable for 8 months	Requires FACS sorting, low scalability; insulin output inferior to human islets; MAFA/UCN3 expression remains immature; dependent on INS‐GFP reporter line	2019/[26]
hESCs and iPSCs	Planar culture and air–liquid interface	Eight‐stage; 47 days	A 10‐chemical cocktail enhances the maintenance of PP cells; up to 82% NKX6‐1^+^/INS^+^ β cells; GSIS and insulin content match human islets; reverses hyperglycemia in mice within 2 weeks and universal for hPSC lines	Overly complex chemical regimen hard to standardize; dynamic secretion not fully characterized; high compound cost	2021/[29]
HUES8 and iPSCs	Planar culture	Six‐stage; >28 days	Actin depolymerization enables 2D planar differentiation; full dynamic GSIS; reverses diabetes in mice within 2 weeks and stable up to 9 months; universal for hPSC lines	Relies on latrunculin A; MAFA/UCN3 expression remains low; SC‐β purity <40% in final product	2020/[36]
hESCs and iPSCs	Planar culture and suspension	Six‐stage; 65 days	Achieves biphasic GSIS; normal electrophysiology and Ca^2+^/cAMP signaling; polyhormonal and enterochromaffin (EC) by‐products reduced; transcriptome approaches adult β‐cell profile; sustains normoglycemia in diabetic mice	Glucose‐mitochondrial coupling remains immature; SC‐β cells exhibit functional heterogeneity; long differentiation cycle (6‐week in vitro maturation)	2022/[30]
hCiPSC	Planar culture and suspension	Six‐stage; 24–26 days	Generates ~60% monohormonal β cells with MAFA/UCN3; robust biphasic GSIS and mature granule ultrastructure; cryopreservable for clinical use	Contains ~15% EC; dynamic secretion and metabolic coupling inferior to primary human islets	2022/[28]

Abbreviations: FACS, fluorescence‐activated cell sorting; GSIS, glucose‐stimulated insulin secretion; hESCs, human embryonic stem cells; hPSCs, human pluripotent stem cells; iPSCs, induced pluripotent stem cell; TGF, transforming growth factor β.

### 2.2 Generating pancreatic progenitor cells from hPSCs

hPSC‐derived pancreatic progenitor cells (PPs) and insulin‐secreting cells have emerged as a promising therapeutic avenue for diabetes. Accumulating evidence demonstrates that PPs derived from hPSCs into mice can give rise to glucose‐responsive, insulin‐producing cells, which can prevent or even reverse diabetes in these animal models.^21^, ^37^, ^38^, ^39^ All adult pancreatic cells originate from PPs, co‐expressing PDX1, PTF1A, NKX6.2, NKX6.1, SOX9, and HNF6.^40^ Notably, only the population co‐expressing PDX1 and NKX6.1 can differentiate into insulin‐secreting β‐cells, whereas PDX1^+^/NKX6.1^−^ progenitors differentiate into dysfunctional polyhormonal cells.^39^, ^40^, ^41^ Inducible factors such as NOGGIN, KGF(FGF7)/FGF10, RA, and EGF have been shown to effectively promote PPs specification.^42^ Nostro et al.^43^ demonstrated that EGF combined with nicotinamide enhances NKX6.1^+^ progenitor cell expression. Furthermore, they also observed that the duration of exposure to RA, FGF10, BMP, and hedgehog signaling inhibitors critically influenced the NKX6.1^+^ cell population, abbreviated induction favored NKX6.1^+^ progenitor specification, whereas prolonged induction promoted polyhormonal cells. Memon et al.^44^ developed an adherent differentiation protocol involving definitive endoderm formation, replating at reduced cell density, and extended induction with RA and FGF10, achieving as high as 90% PDX1^+^/NKX6.1^+^ pancreatic progenitor cells. Moreover, the group has found a population of PDX1^+^/NKX6.1^−^ pancreatic cells capable of further differentiating into functional β cells, whose transcriptome closely resembled native β cells.^45^ Jiang et al.^46^ used a growth factor‐free protocol to generate DE and PPs from hPSCs, thereby circumventing the high cost and compromised cell associated with viability of conventional activin A‐based approaches. A principal challenge in PP‐based strategies remains their scalable expansion, as maintaining stable co‐expression of PDX1 and NKX6.1 during prolonged culture has proven difficult.^47^, ^48^, ^49^ It is worth noting that the BET inhibitor I‐BET151 emerged as a significant advance: it potently promotes the expansion of PDX1^+^/NKX6.1^+^ progenitors (ePPs), which maintain a stable progenitor phenotype long term and retain the capacity to differentiate efficiently into functional β cells.^50^

### 2.3 Construction of islet‐like organs from hPSCs

Islet‐like organs are 3D constructs derived from primary stem cells or pancreatic tissue that recapitulate the structural and functional characteristics of native islets. Native islets contain five hormone‐secreting cell types (α, β, δ, ε, and PP cells) that act in concert to maintain glucose homeostasis.^51^ Kim et al.^52^ developed a multistep protocol to differentiate hESCs into pancreatic endocrine cells (PECs), which subsequently self‐organized into uniformly sized islet‐like clusters (100–150 μm in diameter). These PECs showed enhanced GSIS in vitro and exhibited responsiveness to potassium channel blockade. Wang et al.^53^ employed a collagen/Matrigel (C–M) bionic scaffold to generate hESC‐derived islet‐like organoids with structural and functional resemblance to mature adult islets. Yoshihara et al.^54^ generated human islet‐like organoids (HILOs) from iPSCs and identified noncanonical WNT4 signaling as a critical regulator of metabolic maturation, conferring robust GSIS. Wang et al.^55^ constructed islet organoids containing β cells, α‐like cells, δ‐like cells, endocrine progenitor cells, and intestinal chromatin‐like cells from chemically induced pluripotent stem cell–derived islet (CiPSC‐istet), with the proportion of β cells reaching 60%. Furthermore, they constructed endocrine subtype‐complete islets comprising five secretory cell types, which can efficiently respond to fluctuations in blood glucose concentrations and possess both glucose‐lowering and glucose‐raising capabilities.^56^ In a related investigation, Wu et al.^57^ successfully differentiated patient‐derived pancreatic endodermal stem cells (EnSCs) into islet‐like tissues (E‐islets), which closely recapitulated primary human islets with respect to morphology, cellular composition, gene expression, and in vitro function. Given the importance of vasculature in glucose sensing and insulin secretion, Jun et al. co‐cultured stem cell‐derived islet cells with endothelial cells and fibroblasts in a fibrin gel to create vascularized islet organoids. Compared to nonvascularized islets, the vascularized islets exhibited a higher proportion of glucose‐ and exendin‐4‐responsive β cells.^58^ Citro et al.^59^ reported a novel biofabrication strategy for constructing functional vascularized islet organs (VIOs) using decellularized lung matrices as scaffolds in conjunction with human umbilical vein endothelial cells and islet cells. The lung matrix's dual‐lumen design allows distinct seeding of endothelial and islet cells, facilitating vascular network integration with islet microenvironments through a 7‐day in vitro perfusion culture. Organoids can also be derived from adult stem cells through self‐renewal and differentiation under suitable in vitro culture conditions. However, whether stem cells exist in adult pancreatic islets has long been controversial.^60^, ^61^, ^62^, ^63^ In 2020, Wang et al.^64^ identified a Procr‐expressing cell population as adult islet stem cells within mouse pancreatic islets. Leveraging these Procr^+^ stem cells, the authors established a 3D co‐culture system with vascular cells and successfully generated functional islet‐like organoids.

### 2.4 Insulin‐producing cells are obtained from other cells in the pancreas

Regenerative medicine has increasingly focused on the reprogramming of adult cells as a strategy for tissue repair, representing a viable pathway toward novel clinical therapies. The pancreas comprises both endocrine and exocrine components. The exocrine component consists predominantly of acinar and ductal cells. A seminal study published in *Nature* first demonstrated that acinar cells are amenable to transdifferentiation into insulin‐producing β cells using three reprogramming factors (M3): Ngn3, Pdx1, and Mafa. However, the efficiency remained limited, and the reprogrammed cells survived only briefly in vivo (<2 months).^65^ To overcome this limitation, Li et al.^66^, ^67^ developed a tandem expression system that markedly enhanced transdifferentiation efficiency, yielding stable, functionally competent pancreatic β cells in vivo. It is also worth noting that these β cells reversed hyperglycemia in diabetic mice. The same group subsequently demonstrated that acinar cells could be directed toward α‐, β‐, and δ‐like endocrine cells through distinct reprogramming factor combinations. These reengineered islet cells were structural and functional copies of native islands and were capable of reversing hyperglycemia. Building on these findings, they employed single‐cell transcriptomics to delineate the molecular trajectory of in vivo β‐cell regeneration over the first 10 days through the M3‐induced transdifferentiation model.^68^ Additionally, Furuyama et al.^69^ identified plasticity in human pancreatic non‐β cells: α and γ cells undergo reprogramming via transcription factors PDX1 and MAFA, gaining glucose‐responsive insulin secretion.

## 3 TYPE 1 DIABETIC ANIMAL MODELS

Animal models play an indispensable role in stem cell–based therapies for diabetes. They serve as essential tools for elucidating the pathophysiology of diabetes, evaluating the safety and efficacy of stem cell interventions and optimizing therapeutic strategies. Commonly used type 1 diabetes models fall into three principal categories: spontaneous diabetic models, chemically induced models, and surgically induced models.

### 3.1 Spontaneously induced T1D animal models

Spontaneously induced diabetic animal models commonly utilized in autoimmune diabetes research include NOD mice, KDP rats, diabetes‐prone BB rats, LEW.1AR1‐iddm (LEW‐IDDM) rats, and Long Evans Tokushima Lean (LETL) rats.^70^, ^71^ The NOD mouse is the most widely used spontaneous animal model of T1D, in which insulitis develops at 3–4 weeks of age and progresses to diabetes. Although its autoimmune pathogenesis closely recapitulates that of human T1DM, the model is inherently constrained by sex‐biased disease incidence, a homogeneous genetic background, and high environmental sensitivity, which collectively limit the direct translation of early intervention findings to newly diagnosed patients and render certain immunological conclusions model specific.^71^, ^72^, ^73^ Characterized by spontaneously developing autoimmune diabetes, the Bio‐Breeder (BB) rat constitutes a well‐established primary model for human T1D. It recapitulates key disease feature, including T‐cell‐mediated pancreatic β‐cell destruction and subsequent insulin‐dependent hyperglycemia.^72^, ^74^ The KDP rat is a key spontaneous model for autoimmune T1D research. Mirroring human T1D, it features autoimmune β‐cell destruction and rapid diabetes onset—independent of age/sex, without significant T lymphopenia, and ~80% develop disease within 220 days. However, the substantial costs associated with its maintenance have impeded its widespread adoption, confining its use largely to genetic and mechanistic investigations.^70^, ^75^ The LETL rat was the first reported rodent model of spontaneous autoimmune β‐cell destruction, with ~20% diabetes incidence.^71^ Its value lies in closely recapitulating key clinical features of human T1D, including acute polyuria, polydipsia, and hyperglycemia.^76^ The LEW‐IDDM rat arises from a spontaneous Iddm8 mutation on chromosome 1 in LEW‐1AR1 rats and develops diabetes through autoimmune β‐cell apoptosis.^77^ The model exhibits two distinct disease‐induction modes: ~2% spontaneous rate and near‐100% via immune perturbation.^78^ Of particular note, unlike most spontaneous models that display pronounced sex bias, the incidence of T1D in LEW‐IDDM rats is distributed approximately between males and females.^79^, ^80^

### 3.2 Chemically induced T1D animal models

Chemical induction represents a widely adopted and cost‐effective approach for establishing diabetic models across diverse animal species. The most commonly employed chemical inducers are streptozotocin (STZ) and alloxan (ALX). The ALX induces hyperglycemia usually in days by producing reactive oxygen species (ROS), which compromise pancreatic β‐cell integrity and ultimately lead to cellular injury and necrosis.^81^ Despite its utility in diabetes research, ALX is associated with several notable limitations, including inadequate model stability, a brief disease window, elevated animal mortality, spontaneous remission, and systemic toxicity.^82^ In contrast, STZ has become the preferred chemical inducer for diabetic model development, attributable to its greater specificity to the pancreatic β cell, and a more favorable toxicity profile.^83^ The effective dosage of STZ is influenced by several factors, including animal species, administration route, and individual physiological status.^70^, ^84^, ^85^ STZ has been employed to induce diabetes across various species, including rodents (rats, mice), rabbits, guinea pigs, and monkeys, each requiring species‐tailored dosing regimens.^86^ High‐dose administration, often achieved via a single intravenous or intraperitoneal injection (100–200 mg/kg in mice; 35–65 mg/kg in rats), results in extensive pancreatic β‐cell destruction and severe insulin deficiency.^83^, ^87^ By contrast, the low‐dose multiple‐injection protocol employs smaller doses (20–40 mg/kg/day) for consecutive days to induce immune‐mediated insulitis.^72^ It is noteworthy that the diabetogenic dose of STZ varies significantly across species: rats typically develop irreversible diabetes with 50 mg/kg or higher, whereas larger animals such as cynomolgus monkeys and pigs require doses around 150 mg/kg. In pigs, transient hyperglycemia may occur initially, with partial correction observed within 4 weeks post‐STZ administration.^86^ Collectively, the STZ‐induced T1D model serves as a predominant preclinical model for efficacy assessment, encompassing antidiabetic therapeutics, insulin formulations, delivery devices, and cell‐based therapies.^88^ However, STZ‐induced diabetes results from direct chemical toxicity to pancreatic β cells and, therefore, fails to reproduce the complex autoimmune microenvironment characteristic of human T1D, which fundamentally undermines its construct validity for evaluating immunomodulatory therapies, transplant immune tolerance, and the immunological outcomes of stem cell–based interventions.

### 3.3 Surgical T1D animal models

Current surgical approaches for inducing diabetes include pancreatectomy, partial pancreatectomy combined with chemical agents, and partial pancreatic duct ligation.^88^ Of these, total pancreatectomy, which entails the removal of both the pancreatic parenchyma and duodenum, is frequently applied in large‐animal preclinical studies such as pigs and rhesus monkeys to model T1D.^89^, ^90^ In rat models, removal of approximately 60% of the pancreas can induce diabetes within 3 months, with similar mechanisms observed in dogs and pigs.^91^ Due to the complexity of the procedures and the associated postoperative complications, the use of this surgical induction approach is considerably limited. Its application is primarily restricted to studies focusing on islet/β‐cell transplantation or investigating the regenerative potential of β cells and their precursors.^88^

## 4 TRANSPLANTATION RESEARCH FOR STEM CELL–BASED THERAPY OF DIABETES

At present, the differentiation protocol of stem cells into pancreatic islet cells has been gradually established and optimized. The results of in vitro functional assessments have laid a solid experimental foundation for cell transplantation. However, significant differences exist between in vitro functions and in vivo therapeutic effects. To further clarify the therapeutic efficacy of differentiated cells within the diabetic microenvironment, it is imperative to comprehensively evaluate their actual therapeutic effects through in vivo transplantation models. This will provide a scientific basis for subsequent clinical research on stem cell–based therapies for diabetes.

### 4.1 Preclinical studies

In vivo transplantation of differentiated PPs or islets cells represents a key strategy for evaluating their functionality, as demonstrated across multiple studies.^23^, ^24^, ^26^, ^27^, ^29^ These findings consistently demonstrate that PPs require substantially longer periods to secrete detectable levels of human C‐peptide posttransplant compared to SC‐β cells. For example, transplantation of pancreatic endoderm cells yielded low C‐peptide concentrations after 30 days, with functional parity in human islets achieved by 3 months.^21^ Similarly, encapsulated endocrine progenitors also required 12 weeks to produce C‐peptide.^92^ In contrast, transplantation of more mature SC‐β cells confers rapid functional outcomes: human C‐peptide levels exceeded 1 ng/mL within 2 weeks,^24^ and glucose‐responsive insulin secretion was already detectable as early as 16 days following transplantation.^27^ Consistent with these results, SC‐β cells reverse hyperglycemia in diabetic mouse within 2 weeks of transplantation.^23^, ^29^

Nonhuman primates (NHPs) serve as the gold‐standard preclinical models for evaluating candidate therapeutics prior to human clinical translation, owing to their extensive genetic, anatomic, metabolic, and physiological similarities with humans.^93^, ^94^ Du et al.^28^ reported that intraportal infusion of hCiPSC‐islets effectively restored endogenous insulin secretion and glycemic homeostasis in diabetic NHPs, with key outcomes including a reduction in HbA1c of >2% and approximately 50% decrease in exogenous insulin requirements. Wu et al.^57^ have shown that when E‐islets are transplanted into diabetic monkeys through the hepatic portal vena, they achieved substantial glycemic control with 3 weeks posttransplantation. However, intrahepatic delivery exposes grafts to liver‐derived stressors, causing loss, functional decline, and impaired hPSC‐islet maturation.^6^, ^95^ In this regard, the anterior rectus abdominis sheath has been identified as a superior implantation site, supporting islet survival and maturation and enhancing C‐peptide secretion to levels fivefold higher than intraportal infusion.^96^

### 4.2 Clinical research on stem cell–based therapy for diabetes

On the basis of the promising preclinical evidence (Table 2), clinical trials have been initiated to evaluate the safety and feasibility of stem cell therapy in diabetic patients. In 2014, ViaCyte initiated a Phase 1/2 trial (NCT02239354) of the encapsulated pancreatic endoderm cells (PEC‐01). Though endocrine cells were detected in grafts 2 years postimplantation, device fibrosis compromised graft function.^97^ In a pivotal Phase 1/2 clinical trial (NCT03163511), the PEC‐01 s were implanted subcutaneously into patients with T1D using the VC‐02 open macroencapsulation device. After transplantation, 35.3% of patients exhibited detectable stimulated C‐peptide as early as 6 months, and approximately 63% of explanted devices showed successful engraftment and insulin expression^98^, ^99^. By using a 2–3‐fold higher cell dose and an improved perforated membrane design, 3 out of 10 patients achieved metabolically relevant C‐peptide levels (≥0.1 nmol/L), accompanied by significantly improved glucose control and reduced insulin requirements. However, therapeutic efficacy was still limited by low β‐cell survival, imbalanced endocrine differentiation, fibrotic host responses, and long‐term dependence on immunosuppression.^100^ A pivotal phase 1 clinical trial (ChiCTR 2300072200) demonstrated the 25‐year‐old women with T1D received transplantation of autologous CiPSC‐islets under the anterior rectus sheath and achieved insulin independence by postoperative day 75, accompanied by markedly improved glycemic control and glucose‐responsive C‐peptide secretion.^55^ Zimislecel, an allogeneic pluripotent stem cell‐derived fully differentiated islet cell therapy, has demonstrated promising efficacy and a favorable safety profile in a phase 1–2 clinical trial (NCT04786262) for T1D. In 14 patients with a minimum 12‐month follow‐up, this therapy successfully restored pancreatic islet function, eliminated severe hypoglycemic events, and significantly improved glycemic control in full‐dose recipients, with 83% of these patients achieving insulin independence.^101^ Endoderm stem cell–derived islets (E‐islets) have shown therapeutic potential for both type 1 and type 2 diabetes. A patient with long‐term type 2 diabetes received autologous transplantation E‐islets and complete insulin withdrawal, durable normalization of glycemic control, and markedly increased C‐peptide secretion (NCT05294822).^57^ Three patients with T1D and severe hypoglycemia underwent autologous and allogeneic E‐islet transplantation. The results showed that autologous E‐islet transplantation achieved significant metabolic improvements only under potent immunosuppression, whereas incomplete immunosuppression led to graft failure due to recurrent autoimmunity. In contrast, allogeneic E‐islet transplantation under full‐dose immunosuppression resulted in durable glycemic control, markedly increased time‐in‐range, reduced HbA1c, restored C‐peptide secretion, and even insulin independence in one patient.^102^

**TABLE 2:** Preclinical studies of stem cell therapy for type 1 diabetes.

Transplant cells	Cell numbers	Animal models	Transplant location	Efficacy and safety	Year/reference
S7 (maturing β cells)	~1.25 × 10^6^ cells/animal	NSG mice	Kidney capsule	Human C‐peptide levels reached >1 ng/mL 2 wpt	2014/[24]
SC‐β cells	5 million SC‐β cells	Immunocompromised mice	Kidney capsule	Insulin secretion detected at 2 wpt	2014/[23]
SC‐β cells	~3–5 × 10^6^ cells	Immunocompromised mice	Renal capsule	Improved glucose tolerance posttransplantation 10 days and maintained until 10 weeks	2019/[27]
Fβ‐cells	1.6 million cells	NOD‐SCID‐γ (NSG) mice	Kidney capsule	Reverse hyperglycemia within 2 weeks; no tumor formation within 20 wpt	2021/[29]
SC‐islets	250–750 SC‐islets	Nondiabetic mice	Kidney capsule	Human C‐peptide detectable at 1 month	2022/[30]
hCiPSC‐islets	3 × 10^6^	Immunodeficient mouse	Kidney capsule	Fasting C‐peptide increased steadily from 2 to 12 wpt remained at ~1 ng/mL up to 36 wpt; no tumor formation	2022/[28]
hCiPSC‐islets	~1 × 10^7^ cells/kg	Diabetic rhesus macaque	Portal vein	Mean HbA1c decreased by >2%; exogenous insulin requirement reduced by 49% at 15 wpt; no tumor formation	2022/[28]
hCiPSC‐islets	40 000–48 000 IEQ/kg	Rhesus macaques	Anterior rectus sheath	C‐peptide reached ~2.0 ng/mL at 8 wpt; HbA1c decreased by 44% at 12 wpt. No tumorigenesis	2023/[96]
E‐islets	6000/30 000 IEQ	Cynomolgus monkey	Intrahepatic	Blood glucose normalized at 3 wpt; no teratoma found	2024/[57]

Abbreviation: wpt, weeks post‐transplantation.

## 5 OVERCOMING IMMUNE REJECTION

Current clinical trials of stem cell–based therapies for diabetes have demonstrated promising efficacy in restoring glycemic control and achieving insulin independence.^55^, ^57^, ^98^, ^99^, ^101^, ^102^ However, these grafts, like other allogeneic transplants, face the persistent challenge of immune rejection.^103^, ^104^ Two principal strategies have been proposed to address this challenge.

### 5.1 Biomaterial‐based islet encapsulation strategies

The immune isolation strategy, commonly referred to as cell encapsulation, employs physical barriers to protect allogeneic primary islets or SC‐islets from host immune surveillance, enabling allogeneic transplantation without systemic immunosuppressants.^105^, ^106^, ^107^ Pioneering work demonstrated that alginate‐encapsulated islets transplanted into diabetic rats conferred superior glycemic control relative to nonencapsulated grafts.^108^

Encapsulation systems are constructed from semipermeable biomaterials that physically sequester islets, excluding immune cells and cytokines while permitting the diffusion of glucose, insulin, nutrients, and other essential small molecules necessary to sustain islet viability and function.^109^ Islet encapsulation has been broadly categorized into three tiers: macroencapsulation, which houses hundreds or thousands of islets within a single device; microencapsulation, which encloses single islets or small groups in semipermeable microcapsules; and nanoencapsulation, which targets individual islets.^110^, ^111^ Macrocapsule devices are fabricated as hollow fibers or hollow membrane‐based constructs and are classified as intravascular or extravascular based on their degree of blood contact status.^112^ Intravascular devices house large numbers of islets within semipermeable hollow fibers that interface directly with host vasculature; this direct hemoperfusion ensures sufficient oxygen and nutrient delivery to encapsulated islets, enhancing their survival while conferring immune protection to the membrane.^113^ A California research group recently developed an improved intravascular device using silicon nanoporous membranes, which confer enhanced hydraulic permeability and precise pore size selectivity to mitigate thrombus formation.^114^ Wang^115^ reported an encapsulation system with tapered nanoporous conduits designed to improve mass transport in a NHP model without immunosuppressive therapy. Furthermore, encapsulating cynomolgus monkey islets in an optimized biomaterial and transplanting them into NHPs' omental pouche preserved allogeneic islet survival and glucose responsiveness for 4 months without immunosuppression.^116^ Extravascular devices contain numerous islets within tubular or planar chambers, avoiding direct contact with blood. Typically fabricated from polyacrylonitrile/PVC copolymers, their porous surface enhances biocompatibility and transplant survival.^117^, ^118^ Although these devices are easier to implant and retrieve than intravascular counterparts, they are susceptible to limited oxygen diffusion and fibrotic encapsulation, both of which impair islet function.^113^, ^119^ The TheraCyte is a double‐membrane extravascular device. Elliott et al.^120^, ^121^ demonstrated that encapsulated neonatal porcine islets successfully reversed diabetes in mice for 16 weeks and maintained viability for 8 weeks in monkeys. ViaCyte's PEC‐Encap device, a single‐layer membrane with 0.45‐μm pores, protected pancreatic progenitors from alloimmune rejection in mice for 30 days^122^ and maintained normoglycemia in immunodeficient models.^92^

Microencapsulation provides a high surface area‐to‐volume ratio that facilitates nutrient exchange.^123^ Alginate remains widely used owing to its mild gentle gelling and low propensity for fibrotic encapsulation.^124^ Transplanted into the peritoneal cavity of mice, alginate‐encapsulated iPS‐derived islet‐like cells showed better function than subcutaneous grafts, where foreign body reactions limited efficacy.^125^ Incorporating the chemokine CXCL12 into alginate microcapsules enhanced insulin secretion and reduced fibrosis, enabling glycemic correction for over 150 days in immunocompetent mice.^126^ Similarly, SC‐β cells encapsulated in modified alginate (TMTD) maintained normoglycemia for over 70 days with minimal fibrosis.^127^

### 5.2 Hypoimmunogenic hPSCs

Although hESCs can partially evade CD8^+^ T‐cell recognition, their differentiated derivatives are still recognized and attacked by T cells.^99^, ^100^, ^128^, ^129^ Furthermore, even autologous iPSCs may trigger immune rejection and be identified by T cells or autologous natural killer (NK) cells.^55^, ^57^, ^130^, ^131^, ^132^ HLA class I molecules (i.e., A, B, and C alleles) present antigenic peptides to CD8^+^ cytotoxic T lymphocytes, whereas HLA class II molecules elicit the activation of CD4^+^ helper T cells. One immune evasion strategy targeting HLA involves the simultaneous knockout of B2M and CIITA, both of which are critical for HLA‐I and HLA‐II expression and can help evade killing by CD8 or CD4 T cells.^133^, ^134^ However, when target cells (such as certain stem cells or cancer cells) lack or weakly express MHC class I molecules, natural killer (NK) cells are activated and kill the target cells.^135^ Xu et al.^136^ applied CRISPR‐Cas9 to generate “pseudo‐homozygous” iPSCs by knocking out a single HLA‐A/B allele in heterozygous lines while retaining HLA‐C. This suppressed CD8^+^ T‐cell proliferation and NK cell activity in vitro and prolonged graft survival in vivo. Additional *CIITA* knockout ablated HLA class II, blocking CD4^+^ T‐cell responses. Subsequently, several research groups have successively established B2M‐knockout iPSC lines and validated their capacity to evade CD8^+^ T‐cell‐mediated immune surveillance.^137^, ^138^ Deuse et al.^139^ generated hypoimmunogenic mouse and human iPSCs by knocking out *B*_*2*_ *M* and *CIITA* and overexpressing *CD47*, enabling prolonged survival in fully allogeneic hosts without immunosuppression. Han et al.^140^ used multiplex CRISPR/Cas9 editing to ablated HLA‐A/‐B/‐C and *CIITA* (to eliminate HLA class I/II expression) in hPSCs, and targeted integration of PD‐L1, HLA‐G, and CD47 into the AAVS1 locus, which blunted T‐cell responses, inhibited NK cell cytotoxicity, and minimized macrophage engulfment in vitro and in vivo while retaining pluripotency and differentiation potential. Gerace et al.^141^ showed that HLA knockout or PD‐L1/HLA‐E overexpression alone only modestly delayed rejection. However, engineering SC‐islets to express IL‐10, TGF‐β, and modified IL‐2 (N88D) recruited regulatory T cells (Tregs) and suppressed effector T cells and NK cells, achieving up to 8‐week graft survival and diabetes reversal in NOD mice. Combined elimination of HLA class I/II and overexpression of CD47 (B2M^−/−^CIITA^−/−^CD47^+^) created immunodeficient pluripotent stem cells in mice and humans. Their differentiated islet cells survived for 4 weeks in immunocompetent allogeneic humanized diabetic mice and improved glycemic control.^142^ Similarly, Hu et al.^90^ applied this strategy to rhesus macaque primary islet cells. After transplantation into cynomolgus monkeys without immunosuppression, serum C‐peptide normalized within 1 week and remained stable for over 6 months, achieving tight glucose control and insulin independence without rejection. Tsuneyoshi et al.^143^ combined B2M knockout with the triple overexpression of HLA‐G, PD‐L1, and PD‐L2, generating an iPSC line capable of evading both innate and adaptive immunity simultaneously. A recent study employed gene‐editing strategies to enable stable overexpression of eight key immunomodulatory genes in hPSCs, namely PD‐L1, CD47, CD200, H2‐M3, FasL, Serpinb9, CCL21, and MFGE8. This modification successfully induced broad‐spectrum immune evasion, providing both theoretical and practical support for the development of off‐the‐shelf cell therapies.^144^ In a first‐in‐human study, CRISPR‐Cas12b‐edited hypoimmune platform (HIP) islet cells (B2M/CIITA knockout plus CD47 overexpression) were transplanted into a long‐standing T1D patient without immunosuppression. At 12 weeks, HIP islets evaded immune rejection, maintained stable glucose‐responsive insulin secretion with detectable C‐peptide, and caused no serious adverse events, validating hypoimmune engineering for allogeneic islet transplantation.^145^

## 6 CONCLUSIONS AND PERSPECTIVES

Although significant advances have been made in the directed differentiation of pancreatic β cells from hPSCs (Table 1), current protocols still face multiple challenges. First, the functional immaturity of SC‐β cells remains a critical bottleneck: incomplete biphasic dynamics of GSIS and delayed development of mitochondrial metabolic pathways.^30^ Second, the final differentiated products exhibit high cellular heterogeneity, insufficient purity, and functional variability across different batches.^146^, ^147^, ^148^ Meanwhile, existing protocols are difficult to scale up for industrial production and are associated with high costs.^149^ Future research should focus on elucidating the spatiotemporal molecular regulatory network governing β‐cell maturation, for instance, remodeling ceramide homeostasis^150^ and regulating Ca_2_^+^homeostasis^151^ facilitate β‐cell maturation. In addition, integrating single‐cell multiomics to guide the optimization and quality control of precise differentiation trajectories, leveraging technologies such as single‐cell RNA sequencing to map the in vivo developmental atlas of the pancreas,^152^, ^153^ delineate the molecular characteristics of distinct cell states during the differentiation of PSCs into β cells,^146^ identify changes in key transcription factors and signaling pathways,^154^, ^155^, ^156^ and develop more accurate and specific biomarkers.^146^

The clinical trials demonstrate that stem cell–derived islet transplantation represents a transformative and clinically feasible therapeutic approach for T1D or T2D with severe endogenous β‐cell failure (Table 3). However, personalized stem cell therapy is associated with high costs and limited accessibility. Autologous cell products require patient‐specific reprogramming, directed differentiation, and rigorous quality assessment, resulting in prolonged manufacturing timelines and substantial financial burden, which consequently restrict their clinical application to highly specialized medical centers.^157^ Furthermore, the lack of insurance coverage for experimental cell therapies in most healthcare systems further restricts patient access.^158^ Patient selection and clinical eligibility criteria represent equally critical determinants of therapeutic outcomes. The majority of clinical studies have enrolled highly selected patient populations that may not be representative of the broader diabetic population. Furthermore, most published clinical reports are characterized by relatively short follow‐up durations and limited cohort sizes, such that long‐term clinical outcomes have yet to be comprehensively and unambiguously defined. Extended follow‐up protocols with standardized endpoints, including HbA1c, C‐peptide levels, insulin independence rates, and adverse event surveillance, are urgently needed to establish the long‐term benefit–risk profile of these therapies.^159^ In summary, although PSC‐based therapies represent a transformative potential approach to diabetes treatment, their path to broad clinical adoption requires coordinated progress across manufacturing scalability, equitable access, ethical governance, rigorous patient selection, and comprehensive long‐term monitoring frameworks.

**TABLE 3:** Clinical trials on pluripotent stem cell–based therapy for diabetes.

Cell type	Number of cells	Transplant site	Immunosuppression	Patient condition	Key clinical outcomes	Year/reference
Allogeneic PEC‐01	6–8 × 10^6^ cells; 90–120 × 10^6^ cells	Subcutaneous (device)	Antithymocyte globulin; tacrolimus; mycophenolate mofetil	15 patients (7 males, 8 females), T1D for >5 years	Increased fasting and glucose‐stimulated C‐P; meal‐stimulated insulin secretion	2021/[98]
Allogeneic PEC‐01	~75 × 10^6^ cells/unit; ~6 × 10^6^ cells/unit	Subcutaneous (device)	Antithymocyte globulin; etanercept; tacrolimus; mycophenolate mofetil	17 patients (9 males, 8 females), T1D for >8 years	35.3% (6/17) patients achieved C‐peptide positivity at 6 months	2021/[99]
Allogeneic PEC‐01	~75 × 10^6^ cells; ~7 × 10^6^ cells	Subcutaneous (device)	Antithymocyte globulin; mycophenolate mofetil; tacrolimus	10 patients with T1D for >5 years	4/10 patients increased time‐in‐range, lowered HbA1c/GMI, and insulin reduction	2024/[100]
Autologous E‐islets	1.2 × 10^6^ IEQs/kg	Hepatic portal vein	Mycophenolate mofetil; tacrolimus	A 59‐year‐old man, T2D for 25 years	Insulin withdrawal; TIR 100%; HbA1c 4.6%	2024/[57]
Autologous CiPSC‐islets	19 843 IEQs/kg	Abdominal anterior rectus sheath	Basiliximab; etanercept; tacrolimus; mycophenolate mofetil	A 25‐year‐old woman, T1D for 11 years	Insulin independence at d75; TIR >98%; HbA1c normalized	2024/[55]
Zimislecel/VX‐880	0.4 × 10^9^ cells; 0.8 × 10^9^ cells	Portal vein	Glucocorticoid‐free immunosuppressive	14 T1D patients age 18–65 years: insulin dependence >5 years	Detectable C‐P; full‐dose group: no severe hypoglycemia (d90–365); all HbA1c <7% (d120); 83% insulin independence (d365)	2025/[101]
Autologous E‐islets	1.2 million IEQs	Hepatic portal vein	Basiliximab; mycophenolate/anti‐inflammatory medication; tacrolimus; mycophenolate mofetil	A 30‐year‐old woman, T1D for 18 years	First failed; second improved TIR/C‐P; lost after noncompliance	2026/[102]
Allogeneic E‐islets HLA fully mismatched	600 000 IEQs	Hepatic portal vein	Anti‐inflammatory medication; tacrolimus; mycophenolate mofetil	A 45‐year‐old man, TID for 4 years	Complete insulin independence by week 36	2026/[102]
Allogeneic E‐islets HLA partially mismatched	1.6 × 10^6^ IEQs	Hepatic portal vein	Anti‐inflammatory medication; tacrolimus; mycophenolate mofetil	A 15‐years‐old woman, T1D for 5 years	Insulin and C‐P reached normal thresholds; markedly reduced insulin	2026/[102]

Abbreviations: C‐P, C‐peptide; IEQ, islet equivalents; T1D, type 1 diabetes; Zimislecel/VX‐880, allogeneic stem cell–derived islet‐cell.

Immune rejection remains one of the foremost obstacles in cell transplantation therapy, and it is particularly critical in immune‐mediated T1D, a condition characterized by the autoimmune destruction of pancreatic β‐cells.^55^, ^57^, ^102^ SC‐β cells may be recognized and targeted by residual or reactivated autoreactive immune cells present within the recipient, ultimately causing transplant failure.^160^ Cell encapsulation and genetic engineering have emerged as two promising strategies to circumvent these immunological barriers; however, each approach, when employed independently, is associated with distinct and unresolved limitations. With respect to encapsulation, hypoxia‐induced cellular necrosis within encapsulation devices, particularly in the central region of macroencapsulation devices. Furthermore, these devices are unable to prevent the transmembrane penetration of soluble pro‐inflammatory cytokines, including IL‐1β, TNF‐α, and IFN‐γ, which continue to exert cytotoxic effects on encapsulated β cells, and the physical barrier inherently impedes direct vascular integration between the graft and host vasculature.^161^, ^162^ For genetic engineering, off‐target effects associated with CRISPR‐based genome editing may introduce genomic mutations, and the long‐term safety remains to be systematically evaluated.^162^ Moreover, hypoimmunogenic cells engineered to evade alloimmune recognition may concurrently attenuate host immunosurveillance against malignant transformation, thereby potentially elevating oncogenic risk.^163^ Future research should focus not only on enhancing the efficacy of each individual strategy but also on rigorously exploring their combinatorial integration. Systematic validation of long‐term safety and therapeutic efficacy in large animal models and well‐designed clinical trials will be indispensable prerequisites for ultimately achieving immunosuppression‐free cell replacement therapy for diabetes.

## AUTHOR CONTRIBUTIONS

**Zifan Li:** Conceptualization; writing – original draft; writing – review and editing. **Yu Kang:** Writing – review and editing. **Yuyu Niu:** Conceptualization; funding acquisition; writing – review and editing.

## FUNDING INFORMATION

This study was funded by National Key R&D Program of China (2021YFA0805700, 2021YFA1102000), the National Natural Science Foundation of China (U2102204), and the Natural Science Foundation of Yunnan Province (202102AA100053, 202403AH310053), Xingdian Talent Support Plan of Yunnan Province, China (202405AB350001).

## CONFLICT OF INTEREST STATEMENT

Yuyu Niu is a member of the associate editors of *Animal Models and Experimental Medicine* (AMEM) and the corresponding author of this article. To minimize bias, he was not involved in any editorial decisions related to the acceptance of this manuscript.

## ETHICS STATEMENT

This article is a literature review based on existing publications and does not involve any new studies with human participants or animals performed by any of authors. Therefore, ethical approval and informed consent are not applicable.
